# Research advances in the molecular classification of gastric cancer

**DOI:** 10.1007/s13402-024-00951-9

**Published:** 2024-05-08

**Authors:** Dike Shi, Zihan Yang, Yanna Cai, Hongbo Li, Lele Lin, Dan Wu, Shengyu Zhang, Qingqu Guo

**Affiliations:** 1 Department of Gastrointestinal Surgery, The Second Affiliated Hospital of Zhejiang University School of Medicine, Jiefang Road, Hangzhou, 310009 China; 2 Department of Gastroenterology, State Key Laboratory of Complex Severe and Rare Diseases, Peking Union Medical College Hospital, Chinese Academy of Medical Sciences and Peking Union Medical College, Beijing, 100730 China

**Keywords:** Gastric cancer, Immune checkpoint inhibitors, Molecular mechanisms, Precision medicine, Prognosis

## Abstract

Gastric cancer (GC) is a malignant tumor with one of the lowest five-year survival rates. Traditional first-line treatment regimens, such as platinum drugs, have limited therapeutic efficacy in treating advanced GC and significant side effects, greatly reducing patient quality of life. In contrast, trastuzumab and other immune checkpoint inhibitors, such as nivolumab and pembrolizumab, have demonstrated consistent and reliable efficacy in treating GC. Here, we discuss the intrinsic characteristics of GC from a molecular perspective and provide a comprehensive review of classification and treatment advances in the disease. Finally, we suggest several strategies based on the intrinsic molecular characteristics of GC to aid in overcoming clinical challenges in the development of precision medicine and improve patient prognosis.

## Introduction

Gastric cancer (GC) is the third most common malignancy worldwide [1]. In 2020, over 1 million people were diagnosed with GC, and nearly 770,000 people died from GC-related diseases [2–4]. The incidence of GC varies by sex and geographic location, with studies showing greater prevalence in East Asia and in men, who are twice as likely to develop GC as women [5, 6]. The median survival for advanced GC is typically less than 12 months, but early diagnosis and prompt surgical treatment can effectively prolong the overall survival (OS). The 5-year survival rate for advanced GC ranges from 5 to 20% but can be as high as 85–100% with early diagnosis [7–9].

GC can be roughly divided into two types based on tumor location: non-cardiac GC and cardiac GC. Chronic Helicobacter pylori infection is the major risk factor for non-cardiac GC, with approximately 90% of patients with GC exhibiting positive H. pylori serology. Other risk factors include excessive intake of alcohol, tobacco smoking, and consumption of a diet with high levels of sodium and preservatives. In contrast, cardiac GC has two major risk factors, H. pylori infection and gastroesophageal reflux (GER) [10, 11]. Over the past few decades, the incidence of non-cardiac GC has declined globally, likely due to the decreasing rate of H. pylori infection [12]. At the same time, the incidence of cardiac GC has increased slightly, especially in some developed countries, likely due to changes in lifestyle [13, 14].

The Lauren classification and the World Health Organization (WHO) classification are the most widely used histologic classifications for GC. The former, first proposed by Lauren et al. in 1965, divides GC into intestinal and diffuse subtypes [15]. The latter, first published by the WHO in 2010, recognizes four GC subtypes: papillary; tubular; mucinous; and poorly cohesive subtype variants, including signet ring cell carcinoma [16]. Whereas the papillary and tubular subtypes are classified as differentiated, the signet ring cell subtype is classified as undifferentiated, indicating that it is associated with a poor prognosis.

However, these morphological classification systems have neither contributed to GC subtype-specific treatment strategies nor promoted study of the mechanisms of tumor formation or development. Thus, the only major development in the understanding of GC has been the emergence of a variety of molecular classification systems that reveal the molecular pathogenesis and the potential drivers of alterations in GC. To contribute more fully to the understanding of GC, this article provides a comprehensive summary of the molecular characteristics and latest treatment strategies for this disease. By providing further insight into the pathophysiology of GC, this review may help identify promising clinical biomarkers and treatment targets for its various subtypes.

## Development of GC molecular classification systems

To better understand the molecular characteristics of GC, in 2003 Kim et al. used DNA chip technology to classify 390 GC samples into highly inflammatory infiltrative and slightly inflammatory infiltrative subtypes [17]. Based on the degree of diffuse infiltration and malignancy, they further classified the latter into three subtypes: diffuse, slightly malignant, and highly malignant subtypes. In the same year, Tay et al. applied comparative genomic hybridization (CGH), microsatellite instability (MSI) feature typing, and gene expression microarray technology to divide GC into tumorigenic, reactive, and gastric-like subtypes, among which the gastric-like subtype is associated with higher OS [18].

In a 2011 analysis of the gene expression of 37 GC cell lines taken from patients in Singapore, Tan et al. classified GC into genomic intestinal (G-INT) and geneti- cally diffuse (G-DIF) subtypes. When the authors validated this classification in an independent cohort of 152 patients from Singapore and Australia, they found that it strongly correlates with clinical outcome (*P* = 0.04) and that the prognosis of the G-INT subtype was significantly better than that of the G-DIF subtype (hazard ratio [HR], 1.79; 95% confidence interval [CI], 1.28–2.51; *P* = 0.001) [19].

**Fig. 1:**
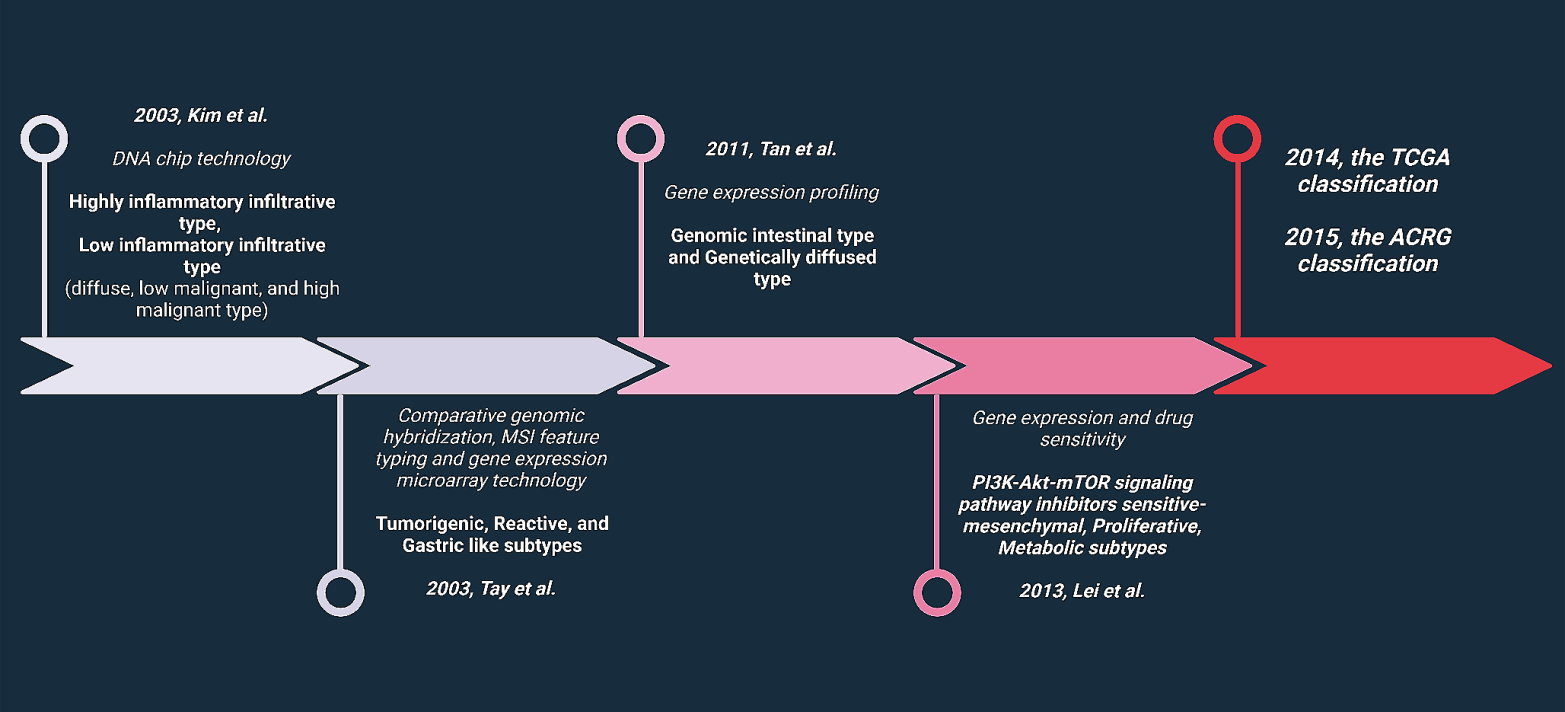
The development of gastric cancer molecular classification systems

In a 2013 analysis of gene expression and drug sensitivity, Lei et al. classified 248 cases of gastric adenocarcinoma into three subtypes. The phosphoinositide 3-phosphorylated Akt-mechanistic target of rapamycin (PI3K-Akt-mTOR) signaling pathway inhibitor-sensitive–mesenchymal subtype, the first subtype, is typically characterized by the pathological feature of tumor stem cell-like heterotypic cells, indicating that drugs targeting the PI3K-Akt-mTOR pathway may be effective treatments for this subtype. The typical molecular characteristics of the proliferative subtype, the second subtype, include genomic instability and a high level of tumor protein p53 (TP53) and of DNA hypomethylation mutations. The third subtype, the metabolic subtype, is particularly sensitive to 5-fluorouracil (5-FU) treatment, resulting in a better prognosis than those of the other two subtypes (*P* = 0.001) [20]. Figure 1 shows the development of the GC molecular classification systems.

## Overview of the cancer genome atlas (TCGA) classification

In 2014, a branch study of The Cancer Genome Atlas (TCGA) project conducted an unsupervised cluster analysis of data obtained from 295 primary tumor tissue samples from patients with gastric adenocarcinoma. The study resulted in the identification of four molecular subtypes: Epstein–Barr virus (EBV)-positive, microsatellite instability (MSI), chromosomal instability (CIN), and genomically stable (GS) subtypes [21]. In 2015, Razvan et al. confirmed this classification through genetic analysis of 251 primary tumor tissues, showing that each subtype is associated with different genomic changes, survival outcomes, and postoperative recurrence patterns, indicating GC heterogeneity [22]. Subsequent correlation analysis of TCGA subtypes and patient prognosis by Bo et al. revealed that patients with resectable MSI and EBV-positive subtypes have better surgical outcomes than patients with the other two subtype. They also observed that patients with the CIN subtype are sensitive to neoadjuvant and/or adjuvant chemotherapy, whereas patients with the GS subtype benefit the least from adjuvant chemotherapy and have the poorest OS [23].

### Epstein–Barr virus (EBV) subtype

EBV is a pathogen associated with various human malignancies, including GC [24, 25]. The EBV-positive subtype is the least common TCGA subtype, accounting for 1.3–28.3% of all GCs worldwide and approximately 75,000 new diagnoses each year [26].

EBV regulates the transformation process of EBV-associated GC (EBVaGC) by expressing various latent genes, such as Epstein-Barr encoding region (EBER), Epstein–Barr nuclear antigen 1 (EBNA1), and Bam-HI A rightward transcripts (BARTs), in host cells [27]. Several researchers have speculated that the low expression of latent membrane protein 2A (LMP2A) by EBV activates the Notch signaling pathway, leading to cancer-cell migration and overexpression of epithelial–mesenchymal transition (EMT) markers [28, 29].

DNA hypermethylation and multi-somatic cell genome mutation are the main characteristics of EBVaGC [27, 30]. Multiple studies have demonstrated that the ascending methylation level of the CpG island (CGI) in the promoter region of cell cycle-related genes, such as cyclin-dependent kinase inhibitor 2A (CDKN2A) and alternate reading frame protein product (p14ARF), particularly in the DNA repair fragments impairs tumor suppression, ultimately causing CIN [31]. Several latent viral proteins, such as EBNA1 and LMP2A, may trigger hypermethylation by promoting overexpression of DNA methyltransferase [32]. LMP2A can also induce methylation of promoter CGI-related genes that help pathogens evade host immune recognition and response by up-regulating DNA methyltransferase-3b (DNMT3b) [33]. Table 1 lists the EBV-latent genes related to GC oncogenesis.

**Table 1:** The EBV-latent genes related to gastric cancer oncogenesis

Biological functions	Related genes	Related downstream molecules/pathways	References
Promote proliferation, cell migration and oncogenesis	EBER	pFAK and pPAK1	[117]
	EBNA1	GKN1 and GKN2 promoters	[32]
	LMP2A	ERK-DNMT3a- AQP3	[116]
	BARF1	NF-κB/cyclin D1	[34]
	BART miRNAs	EBV-miR-BART5-3p/TP53	[35]
Resist apoptosis	LMP2A	NF-κB/survivin	[36]
	BART miRNAs	EBV-miR-BART4-5p/Bid	[37]
	BARF1	Bcl-2/Bax	[38]
Induce chemoresistance	EBER	IL-6-STAT3-p21/p27	[117]
	EBNA1	Compete with TP53 for ubiquitin-specific protease 7 binding	[39]

In multi-somatic cell genome mutation, the representative mutation gene is phosphatidylinositol-4,5-bisphophase 3-kinase, catalytic subunit alpha (PIK3CA) [40, 41], which accounts for 60–80% of the entire EBV-positive GC mutation frequency. AT rich interactive domain containing protein 1A (ARID1A) [42] and BCL6 co-repressor (BCOR) [43] are two other genes in EBV-positive subtypes, found in approximately 45% and 30%, respectively. Whereas the mutation frequency of TP53 in CIN subtypes is approximately 70%, mutations in EBV-related GC are rare. Interestingly, although PIK3CA has historically been classified as an oncogene, elevated PIK3CA expression correlates positively with better 5-year OS in EBV-negative GC but not EBV-positive GC (57.8% vs 33.4%, respectively; *P* *< *0.001) [41].

The clinicopathological characteristics of EBV-positive GC have been extensively examined in recent years. In a 2019 systematic analysis of these characteristics in 1132 patients with EBV-positive GC, Yanagi et al. observed a predominance in the proximal stomach location, particularly in the remnant stomach after subtotal gastrectomy [44]. Survival meta-analyses indicated a higher incidence of EBV-positive GC in young males, although research suggests this gender disparity decreases with age [45–47]. Multivariate factor prognostic analyses using the Cox proportional hazards model showed a median survival of 8.5 years for patients with EBV-positive tumors and 5.3 years for those with EBV-negative tumors (HR, 0.72; 95% CI, 0.61–0.86) [47]. This finding is consistent with the results of a cohort study from the TCGA, indicating that EBV-positive GC is associated with longer recurrence-free survival (RFS) and OS than the MSI, GS, and CIN subtypes [48].

In 2021, Bai et al. developed an EBV algorithm based on next-generation sequencing (NGS) detection of four EBV genes that can accurately identify EBV-positive GC [49]. Using this algorithm, they found that the overall diagnostic accuracy for EBV-positive GC can reach approximately 98.7%. More importantly, they observed that patients with EBV-positive GC with high cytotoxic T-lymphocyte associated protein 4 (CTLA-4) levels were less responsive to single-agent anti-programmed cell death protein 1 (PD-1)/L1 therapy and derived greater benefit from a combination of PD-1/L1 and CTLA-4 blockade than anti-PD-1/L1 monotherapy, with a median progression-free (mPFS) of 8.5 compared with 2.0 months, respectively (*P* *< *0.001). Another study reported that combining anti-PD-1 and anti-T-cell immunoglobulin mucin-3 (TIM-3) monoclonal antibodies (mAbs) directly promoted the immunocompetence of cytotoxic T lymphocytes, suggesting that dual-immune checkpoint inhibition targeting PD-1 and TIM-3 may increase response rates in EBV-positive GC [50]. However, neoadjuvant chemotherapy is not appropriate for patients with EBV-positive GC due to its possible disruption of the protective effect of infiltrating cytotoxic T-(CD8+ T) cells. In a retrospective study, Qiu et al. discovered that the overall response rate of patients with EBV-positive GC receiving first-line chemotherapy was as low as 33% [51]. In accordance, Tong et al. found no significant difference in OS prognosis between patients with EBV-positive GC who received neo-adjuvant chemotherapy and those who did not (*P* = 0.58) [52].

### MSI subtypes

Microsatellite sequences (MSs) are short tandem repeat DNA sequences found in the genome that typically consist of one to six nucleotides arranged in tandem repeats. MSs display a polymorphic distribution due to variations in the type of repetitions within their core repeating units and can be located in non-coding regions. The insertion or deletion of simple repeating units leads to the emergence of new microsatellite alleles, resulting in MSI in MSs [53, 54]. Generally, MSI is caused by mismatch repair deficiency (dMMR), and the direct detection of MSI sequence changes and MMR gene deletion can both confirm the occurrence of MSI. MMR gene-defect detection usually relies on immunohistochemistry of target proteins MutL homolog 1 (MLH1), MSH2, MSH6, and postmeiotic segregation increased 2 (PMS2) [53, 55].

Since first being discovered in colorectal cancer (CRC), MSI has been considered a unique characteristic of hereditary nonpolypic CRC [56]. Subsequently, MSI has been found to exist in various sporadic tumors, including GC [57, 58], lung cancer [59] and endometrial cancer [60]. The molecular pathogenesis of MSI in GC and CRC is markedly different. The main genes causing MSI in CRC are h-MLH1 and h-MLH2, accounting for over 80% of mutations, whereas h-MSH6 causes only approximately 5% of all mutations [61]. In GC, 50% of MSI-associated mutations are caused by methylation of the h-MLH1 promoter, whereas mutations related to both h-MLH1 and h-MLH2 promoters only constitute 10–12% of cases [62].

In addition to high MLH1 methylation levels, two other molecular characteristics of MSI-associated GC include high lymphocyte infiltration and proportional expression of immune checkpoint-related proteins [21, 63]. Therefore, MSI is considered both a separate subtype in TCGA classification and a clinical biological prognostic marker. In a 2010 meta-analysis of 1556 patients with GC (high microsatellite instability [MSI-H] ratio, 7.8%), Guastadisegni et al. found that the 5-year disease-free survival (DFS) rate of patients with MSI was significantly higher than that of patients with microsatellite stability (MSS) (71.8% vs 52.3%; *P* *< *0.001), as well as the 5-year OS rate of patients with MSI was higher than that of patients with MSS (77.5% vs 59.3%; *P* *< *0.001). Their findings indicate that MSI is an independent prognostic factor for DFS and OS in GC [64]. At the same time, they indicate that compared with the treatment of patients with MSS with combination surgery and chemotherapy (DFS rate, 77%; OS rate, 83%), treatment of patients with MSI-H with this combination is less beneficial (DFS rate, 70%; OS rate, 75%), suggesting that chemotherapy may be a detrimental factor for OS in MSI-H GC.

**Fig. 2:**
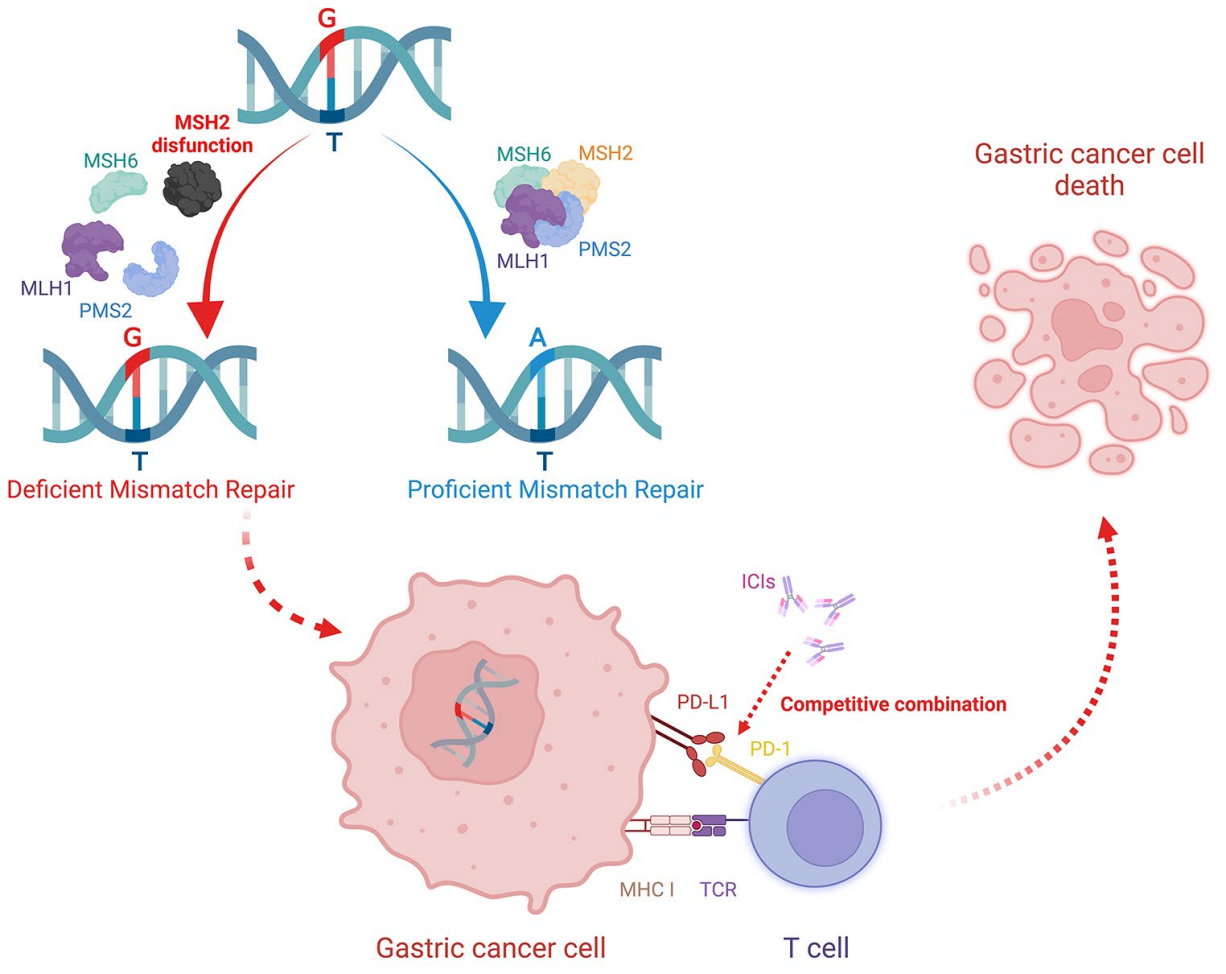
The mechanism of deficient mismatch repair (dMMR) related-microsatellite instability (MSI) in gastric cancer (GC) and the therapeutic mechanism of immune checkpoint inhibitors (ICIs). Mismatch repair proteins, such as MLH1, MSH2, MSH6, and PMS2, correct DNA replication errors, whereas MSH2 dysfunction can impair this process, leading to MSI in GC cells. ICIs such as nivolumab and pembrolizumab can competitively block PD-1/PD-L1 binding, thereby enhancing T-cell-mediated tumor-cell killing

Although patients with MSI-H may not benefit from fluoropyrimidine-containing chemotherapy regimens, recent advances in targeted therapy research have shown that immune checkpoint inhibitors (ICIs) have better efficacy in MSI GC than MSS GC. In a 2021 meta-analysis of 2545 patients with GC with assessable MSI status data, Pietrantonio et al. found that the HR for OS with anti-PD-1-based regimens was 0.34 (95% CI, 0.21–0.54) compared with 0.85 (95% CI, 0.71–1.00) for MSS cancers, as well as that the treatment effect significantly differed between the two subgroups (*P* for interaction = 0.003) [65]. In 2020, Jin et al. reported that after receiving single-dose anti- PD-1 treatment combined with first-line chemotherapy, a patient with unresectable, MSI-H, locally advanced GC achieved complete pathological remission according to computed tomography and histopathology [66]. These studies indicate that patients with MSI-H GC may benefit from immunotherapy and that MSI can serve as a prognostic indicator of the therapeutic efficacy of ICIs in the treatment of GC. Figure 2 shows the mechanism of dMMR-related MSI and the therapeutic mechanism of immune checkpoint inhibitors (ICIs) [67].

### CIN subtypes

The CIN subtype is the most common gastric molecular subtype according to TCGA classification, accounting for approximately 50% of all GCs [26]. The CIN subtype is the most common gastric molecular subtype according to TCGA classification, accounting for approximately 50% of all GCs [68]. Another factor contributing to CIN subtype formation is cell-cycle checkpoint defects caused by the upregulation of expression levels of mitotic checkpoint catalytic subunit 1 (CDK1) and its regulatory factors, cyclin B1/B2, cyclin A, cell division cycle 25 (CDC25), cyclin-dependent kinases regulatory subunit 1 (CKS1), and CKS2. Other contributing factors are mitotic stress and replication stress induced by mutations in oncogenes such as hepatocyte growth factor receptor (c-MET), B-Raf (BRAF), retinoblastoma (RB), TP53, polo-Like kinases (PLKs) and cyclin D1 [69–73].

According to the TCGA Program, approximately 70% of CIN-associated GCs (CINaGCs) exhibit an intestinal phenotype accompanied by P53 mutation [74]. However, Gonzalez et al. showed that immunohistochemical detection of wild-type P53 expression alone is insufficient to distinguish CIN subtypes from GS subtypes, but more accurate molecular markers have not yet been found [75]. It is currently believed that the defining characteristics of CIN subtypes are the amplification of genes encoding receptor tyrosine kinase (RTKs) and the overexpression of cell cycle-related genes. The current study showed that the upregulation of oncogenes such as MET, c-myc (MYC), heparin- binding secretory transforming gene (HST1)/integrator complex subunit 2 (INT2) and ERBB2 is positively correlated with the poor prognosis for CINaGC. Thus, inhibitors targeting molecules related to these oncogenes can be used as therapeutic options for treating CINaGC. Hisamatsu et al. confirmed that the overexpression of epidermal growth factor receptor (EGFR) and phosphorylated Akt (P-AKT) can also lead to DNA aneuploidy (*P* = 0.0002 and *P* = 0.0302, respectively) [76]. Notably, in 2022 Poghosyan et al. found that gastric and bile acid reflux can cause changes in specific chromosome copy numbers in the esophageal cancer cell line CP-A [77], which leads to a surge in the number of cells with abnormal chromosome segregation. This may explain the high incidence of occurrence of CINaGC in the proximal part of the stomach, esophagus, and cardi.

To investigate the impact of adjuvant chemotherapy on CINaGC, Ippolito et al. divided 532 cases of CINaGC into four subgroups: low (n = 97, 18.2%), moderate (n = 214, 40.2%), substantial (n = 161, 30.3%) and high (n = 60, 11.3%) CIN subgroups [78]. A comparison of the corresponding biopsies from before adjuvant chemotherapy and resected tumors after chemotherapy across all 38 patients revealed a consistent classification into the respective CIN groups in only 10 (26%) cases. Of the 28 (73%) cases with discrepancies in CIN classification, 19 cases (68%) were recategorized from a higher CIN subgroup to a lower one and 9 from the high-CIN subgroup to lower subgroups, suggesting that adjuvant chemotherapy may alter the internal molecular characteristics of CINaGC. Notably, the high- CIN subgroup (>75%) had the worst prognosis among the 4 subgroups, and a high level of CIN (>50%) was significantly correlated with abnormal p53 expression (*P* = 0.004).

Currently, no specific therapeutic measures exist for treatment of CINaGC. However, platinum-based adjuvant chemotherapies have been identified as the preferred treatment option for GC adjuvant chemotherapy, and have improved the DFS of patients with intestinal subtype GC. Docetaxel has also shown clinical benefit in undifferentiated intestinal GC [79]. Given that the CIN subtype is induced by recurrent amplifications of genes encoding RTKs, such as EGFR, fibroblast growth factor receptor 2 (FGFR2), human epidermal growth factor receptor 2 (HER2), and MET and cell-cycle mediators, several researchers have suggested that CINaGC may be sensitive to RTK-targeted agents or DNA damaging drugs.

### GS subtypes

Genomically stable associated GC (GSaGC) subtypes mainly invade the gastric antrum or pylorus and account for approximately 20% of all GCs. Unlike CINaGC, GSaGC is typically categorized as the diffuse subtype according to Lauren classification. The two major molecular characteristics of GSaGC are RAS homologous (RhoA) mutation and claudin-18 (CLDN18)–Rho GTPase activating protein (ARHGAP) fusion [21, 80, 81]. A micro-G-protein molecule with GTPase activity in the Ras superfamily, RhoA can activate signal transducer and activator of transcription 3 (STAT3) to promote the formation of various tumors, including breast [82], gastric cancer [83] and colorectal cancer (CRC) [84]. However, RhoA mutations are uncommon and only observed in approximately 16.3–25.4% of diffuse GCs [85, 86]. Hayakawa et al. confirmed that mutated RhoA loses its ability to bind with glutamic pyruvic transaminase (GTP), promoting cell invasion and proliferation in GSaGC [87].

The fusion of CLDN18 with ARHGAP, a GTP enzyme-activating protein involved in the regulation of normal cell-proliferation cycles, is another molecular event associated with the GS subtype that does not typically coexist with RhoA mutations [88]. Located at the 3q22.3 site of human chromosome 3, the CLDN18 gene primarily maintains tight junctions between cells. Its fusion with ARHGAP induces the production of a new gene, CLDN18-ARHGAP26, which contains an almost complete coding region of CLDN18 and a conserved domain region of ARHGAP while preserving the C-terminal GAP domain [89]. This process may affect the regulation of the RhoA pathway and GC cell phenotype by ARHGAP. Moreover, the presence of fusion proteins may disrupt the structure of wild-type CLDN18 protein, subsequently impacting cancer-cell adhesion and promoting tumor migration and invasion.

A functional study conducted by Yao et al. suggested that introducing CLDN18- ARHGAP26 fusion to tumor cells can result in the loss of epithelial phenotype, EMT, and inhibition of the RhoA signaling pathway, thereby contributing to tumor invasiveness in cancer cell lines [89]. Using a murine model, Wang et al. found that CLDN18-ARHGAP26 could activate the PI3K/AKT-mTOR-FAS pathway and stimu- late fatty acid secretion, thus enhancing metabolism and reproduction in Treg cells and leading to the formation of an immunosuppressive tumor microenvironment [90].

The prognosis for GSaGC is relatively poor compared with that of other TCGA subtypes, characterized by high recurrence and a lack of standard therapeutic strategies. However, a 2020 molecular analysis of the tumor immune microenvironment (TIME) at the University of Osaka showed that macrophages and effector B cells are enriched in the GSaGC tumor micro-environment and that approximately 55% of GSaGCs have a tertiary lymphoid structure, providing a theoretical basis for using immunotherapy to treat this subtype [91]. The CLDN18-ARHGAP fusion GC-bearing model constructed by Wang et al. in 2022 revealed that PI3K inhibitors (PI3Kis) can partially reverse the inhibitory TIME induced by CLDN18-ARHGAP26 fusion, thereby inhibiting tumor growth [92].

When Sohn et al. performed subset analysis of patients in the MD Anderson cohort who had American Joint Committee on Cancer (AJCC) stage II, III, or IV GSaGC without distant metastasis (n = 157), they found that GSaGC was associated with no increased clinical benefit from adjuvant chemotherapy (*P* = 0.66) [93]. Shariftabrizi et al. suggested that excessive activation of the RhoA pathway may relate to reduced sensitivity of GC cells to chemotherapy drugs but that miR-31 therapy could partially reverse this reduction [94]. Yoon et al. confirmed that the combination of Rhoa pathway inhibition, 5-FU administration, and cisplatin therapy could decrease the proliferation and metastasis of GC cells to a greater extent than adjuvant chemotherapy monotherapy [95]. These findings indicate that focusing on the cell-adhesion pathway, especially related targets of the RhoA pathway, may provide new perspectives for the systematic treatment of GSaGC.

## Research advances in ACRG classification

After conducting a comprehensive analysis of the gene expression levels of 300 gastric adenocarcinomas in 2015, the Asian Cancer Research Group (ACRG) proposed a new gastric cancer molecular classification that comprised four molecular subtypes: MSI-H, MSS/EMT, MSS/TP53 mutant (MSS/TP53+), and MSS/TP53 wild-type (MSS/TP53–) subtypes [22].

Compared to the TCGA subtypes, the four ACRG subtypes are more closely related to clinical prognosis. The MSI-H subtype (22.3% of GCs) mostly occurs in the gastric antrum (75%), and most cases would be classified into the intestinal subtype by Lauren classification. This subtype often presents with early diagnosis and improved clinical prognosis [96].

Immunotherapy for the MSI-high (MSI-H) subtype is significantly effective. The molecular characteristics of this subtype are similar to those of the MSI subtype of TCGA classification, with excessive methylation of encoding genes, including Kirsten rat sarcoma viral oncogene homolog (KRAS), phosphoinositide 3-kinase (PI3K)- phosphatase and tensin homolog (PTEN)- mtOR pathway, ARID1A, ERBB2, ERBB3, and ALK [97]. The molecular changes in the MSS/EMT (15.3%) subtype, which is typically classified as the diffuse subtype (80%) by Lauren criteria, are closely tied to alterations in cell adhesion and activity, with cadherin-1 (CDH1) gene-deletion expression being a notable trait. The three prominent clinical characteristics of this subtype include younger onset, poor pathological stage at initial diagnosis, and high probability of recurrence and metastasis, typically leading to the worst prognosis among the subtypes [98].

A high incidence of EBV infection characterizes the clinical features of the MSS/TP53+ subtype (26.3%), whose molecular characteristics include activation of the TP53 pathway and high-frequency mutations in genes such as ARID1A, KRAS, PI3KCA, and SMA- and MAD-related protein 4 (SMAD4). The MSS/TP53– subtype (35.7%) has the highest TP53 mutation rate (60%), possibly due to loss of function of the TP53 pathway. The molecular characteristics of this subtype include partial amplification of oncogenes such as mouse double minute 2 homolog (MDM2), roundabout guidance receptor 2 (ROBO2), GATA binding protein 6 (GATA6), MYC, ERBB2, EGFR, cyclin E1 (CCNE1), and cyclin D1 (CCND1). The (MSS)/TP53+ and (MSS)/TP53– subtypes, which are more common in male patients (63%), have an overall clinical prognosis between those of the MSI-H and MSS/EMT subtypes [96, 99, 100]. ACRG classification has clinical significance for two primary reasons. First, it divides GC into 2 distinct categories, MSS and MSI subtypes, of which the TP53 activation level of the MSS subtype serves as an independent typing standard and the MSS/EMT subtypes are associated with poor prognosis. Compared with TCGA classification, ACRG classification clarifies the correlation between GC molecular typing and clinical prognosis. Second, ACRG classification describes the clinical characteristics of each subtype completely differently, which provides for the possibility of implementation of personalized clinical treatment strategies. For example, by allowing for identification of patients with the MSS/EMT subtype, who must undergo systematic intraperitoneal infusion chemotherapy after surgical resection due the high incidence of peritoneal metastasis associated with this molecular subtype, it greatly reduces the probability of tumor recurrence and metastasis.

Despite a high level of similarity between the molecular characteristics of the TCGA MSI subtype and the ACRG MSI-H subtype, the remaining subtypes in the two classification systems do not align well. These differences may stem from GC heterogeneity among disparate populations, as the tissue source sites upon which TCGA classification was based were mainly from the United States [21], whereas the ACRG sourced its data from the Asian population in South Korea [22]. Regional factors in the pathogenesis of GC must be further studied in the future. For now, ACRG classification holds greater relevance for Southeast Asia, which has the highest GC incidence rate in the world, than TCGA classification. Table 2 shows the results of comparison of the TCGA and ACGR molecular classification systems.

**Table 2:** The cancer genome atlas and the asian cancer research group molecular classification systems for gastric cancer

Molecular classification	TCGA classification (2014)	ACGR classification (2015)
Data sources	295 primary GC tumor samples from TCGA database	300 primary GC tumor samples from Samsung Medical Center, Seoul, South Korea
Analysis methods	Whole-genome sequencing, array-based somatic copy number analysis, whole-exome sequencing, array-based DNA methylation profiling, messenger RNA sequencing, microRNA (miRNA) sequencing, reverse-phase protein array, microsatellite instability (MSI) testing; unsupervised clustering, integrative clustering	Whole-genome sequencing, gene expression profiling, genome-wide copy number microarrays, targeted gene sequencing; Principal component analysis
Classification standard	(a) EBV infection (b) MSI high or not (c) Genomically stable or not (d) Chromosomal instability	(a) MSS or MSI (b) EMT (E-cadherin aberrant) (c) P53 aberrant
Subtypes	EBV positive subtype MSI subtype GS subtype CNI subtype	MSI-H subtype MSS/EMT subtype MSS/P53+ subtype MSS/P53- subtype
Clinical value	EBV positive subtype: better surgical outcomes, immunotherapy sensitive; MSI subtype: better surgical outcomes; CIN subtype: neoadjuvant and/or adjuvant chemotherapy sensitive; GS subtype: poor prognosis	MSI-H: better prognosis, immunotherapy sensitive; MSS/EMT: poor prognosis, high recurrence and metastasis rate; MSS/P53+: high EBV infection rate; MSS/P53-: high TP53 mutation rate

*TCGA* the cancer genome atlas program, *ACGR* asian cancer research group, *GC* gastric cancer, *EBV* Epstein–Barr virus, *MSI* microsatellite instability, *MSS* microsatellite stability, *MSI-H* high microsatellite instability, *GS* genomically stable, *CNI* chromosomal instability, *EMT* epithelial-mesenchymal transition

## The future of multi-dimensional GC molecular classification

Although the TCGA and ACGR classification systems provide insight into the heterogeneity of GC and prognostic value to some extent, they fail to meet clinical therapeutic needs. With the development of transcriptomics, proteomics, metabolomics, and machine learning (ML) algorithms over the past 10 years, researchers have constructed a multi-dimensional GC molecular classification map from various perspectives. In 2020, Liu et al. identified immunity-high (IM-H) and immunity-low (IM-L) GC subtypes on based on analysis of 797 immune-related genes. The IM-H subtype shows stronger immune activity, has a worse prognosis, and may benefit from anti-CTLA4 treatment [101]. In a 2021 examination of ferroptosis-related expression profiles and DNA methylation, Xiao et al. characterized three ferroptosis subtypes closely associated with clinical prognosis, chemotherapy impact, and immunotherapy response [102].

In 2022, Lin et al. classified two GC phenotypes according to their exosome-based gene signatures, including glutathione peroxidase 3 (GPX3), regulator of G protein signaling 2 (RGS2), matrilin 3 (MATN3), solute carrier family 7 member 2 (SLC7A2), and synuclein gamma (SNCG), helping to predict tumor responses to an anti-CTLA4 inhibitor [103]. In 2023, Dong et al. categorized 743 stomach adenocarcinoma samples into 3 clusters by mRNA levels of oxidative stress and metabolism-related genes to produce an oxidative stress and metabolism-related gene (OMRG)-based molecular classification system that significantly correlates with immune cells and immune checkpoints [104].

Trastuzumab is a humanized monoclonal antibody targeting HER2 extracellular domain 4, which inhibits downstream signaling activation and cancer cell proliferation. Trastuzumab combined with chemotherapy has been established as the standard first-line treatment for HER2-positive advanced GC [105], but due to primary or acquired resistance to trastuzumab, only a subset of patients benefit from this treatment. Therefore, it is of great interest to establish a classification system that can further guide the use of trastuzumab. By profiling the proteome of 206 gastric tumor samples, Li et al. revealed that patients with high T-cell receptor signaling respond to anti-HER2-based therapy, whereas activation of the extracellular matrix/PI3K-AKT pathway impairs the anti-tumor effect of trastuzumab [106]. but due to primary or acquired resistance to trastuzumab, only a subset of patients benefit from this treatment. Therefore, it is of great interest to establish a classification system that can further guide the use of trastuzumab. By profiling the proteome of 206 gastric tumor samples, Li et al. revealed that patients with high T-cell receptor signaling respond to anti-HER2-based therapy, whereas activation of the extracellular matrix/PI3K-AKT pathway impairs the anti-tumor effect of trastuzumab [107]. By performing whole exome sequencing (WES) on paired tumor tissues from 23 patients with GC before trastuzumab treatment at baseline and at progressive disease (PD), Xu et al. identified the most common genes mutations (AURKA, MYC, STK11, and LRP6) associated with failure of anti-HER2 therapy [108].

Nebuliumab, a monoclonal antibody that can relieve the suppression of immune response mediated by the PD-1 pathway and restore tumor-specific T-cell immunity, has been approved in various countries for first- and third-line treatment of unresectable/metastatic GC [105]. By conducting transcriptomic profiling of 36 MSI-H/dMMR gastrointestinal tumors to identify predictors of response to PD-1 blockade, Chida et al. found that vascular endothelial growth factor (VEGF-A) was significantly correlated with enriched pathways in nonresponders [109]. In a study of the tumor microenvironment (TME) of 2456 patients with GC, Chen et al. classified 4 subtypes—TMEclassifier-A, B, C, and D—and discovered that patients with the TMEclassifier-B subtype without chemotherapy benefit responded best to pembrolizumab treatment (PD-1 inhibitor). Patients with the TMEclassifier-A subtype responded to pembrolizumab less strongly than those with the TMEclassifier-B subtype, and patients with the TMEclassifier-C and TMEclassifier-D subtypes responded poorly to immunotherapy [110].

As described above, from ferroptosis to exosome analysis, from TIME to targeted drug response investigation, techniques using multiple omics (multiomics) have been widely applied to GC molecular classification in recent years. These new classifications not only greatly enrich our understanding of the underlying mechanisms of GC but also aid clinicians in the selection of novel molecularly targeted drugs. Nevertheless, the recently published studies faced several limitations. First, despite the fact that current classification systems were established from multiple perspectives, each system relies on different data sets collected from patients with distinct demographic and clinical characteristics, including race, world region, and tumor stage. Therefore, the lack of comparability between different classification systems makes it difficult to integrate multiomics data. Second, most classification systems still require prospective research data for verification, which are often difficult to obtain. Third, investigation of the practical application of these systems remains crucial, particularly that of drug-response-oriented classification systems. The study design of such investigations should consider tumor stage and aim to predict drug resistance and derive therapeutic protocols that maximize patient benefit. Finally, the existing classification systems are mainly based on detection of genetic molecules, which is a highly technically demanding and expensive endeavor that yields results often difficult to apply and commercialize.

Naturally, development of the next generation of molecular classification systems for GC must focus on several considerations. First, the multiomics signatures of GC should be generated from relatively large and comparable GC data sets. ML algorithms based on the development of transposed convolution network theory may help identify patterns among a vast quantity of molecular data and draw comprehensive conclusions. Li et al. has proposed development of a refined molecular classification using multiomics data and integrating optimal algorithms, similarity network fusion, and consensus-clustering methods. In this way, an extreme gradient-boosting ML prediction model can be developed that may provide a practical subtyping framework to improve the treatment of GC [111]. Moreover, in the future, clinicians also require more effective molecular classification systems that can guide the choice of treatment options, including surgy, chemotherapy, and/or immunotherapy, for patients at different stages. From the perspective of clinical practicality and cost effectiveness, it may be more essential to identify several key biomarkers that can distinguish treatment responsiveness among the GC subgroups than to comprehensively portray the inherent molecular differences among the different types of GC.

## Discussion

GC is a highly heterogeneous disease driven by multiple gene mutations and epigenetic anomalies for which systemic treatment plays a crucial role in its multidisciplinary management. Nevertheless, cytotoxic drugs, radiotherapy, and immunotherapy provide limited clinical benefit, and drug resistance is a major factor leading to therapy failure. Therefore, in addition to traditional classification by morphology, classification by identification of tumor heterogeneity and molecular characteristics is vital for improving treatment efficacy. With the rapid development of genomics, GC molecular typing as represented by ACRG and TCGA criteria can effectively predict the response of different molecular subtypes to systemic therapy, helping maximize therapeutic benefit while avoiding toxic side effects.

In addition to providing potential therapeutic targets, molecular classification and profiling of GC can generate several prognostic and predictive biomarkers. Analysis of the data collected to develop the TCGA and ACRG classification systems not only revealed the molecular and etiologic differences across various subtypes but also yielded many potentially targetable genomic alterations. Recently, researchers have begun using promising new technologies to depict the molecular signatures of GC from multiomics dimensions. The development of advanced ML algorithms; the integration and evolution of genomic, transcriptomic, proteomic, metabolomic, and spatial transcriptomic techniques; and advances in single-cell sequencing all portend the ability to amalgamate disparate multi-dimensional and multiomics data for deeper exploration of the malignant phenotypes and internal molecular characteristics of GC. Compared with the TCGA and ACGR classification systems, classifications based on these data will emphasize the differentiation of prognosis and therapeutic response among different subtypes, increasing clinical translational potential.

Preclinical and clinical investigations have been conducted into therapeutic agents that target molecular alterations defined by the subtyping and profiling of GC. These studies will also aid in identifying prognostic and predictive biomarkers of tumors by correlating molecular profiles with clinical outcomes, such as OS, PFS, DFS, and tumor response. Analysis of larger data sets in these investigations will help optimize the chemotherapy regimen for individual patients and facilitate the development of novel targeted therapies.

Immunotherapy has shown promising efficacy in treating various solid tumors, includ- ing GC. Using existing molecular classifications of GC to screen potential beneficiaries of immunotherapy is expected to improve therapeutic effect. In TCGA classification, the MSI-H- and EBV-positive subtypes, although both sensitive to immunotherapy, do not produce identical immune responses, calling for investigation of the correlation between subtype and immunotherapy response. In terms of clinical application, proteomics and microRNA analysis as well as plasma circulating-tumor DNA detection may be more cost-effective ways to identify therapeutic predictors [112–114].

Studies of several newly developed molecular classification systems have reported predictive value and HER2 or PD-1-targeted immunotherapy response and identified drug-resistance-associated molecules and signaling pathways. However, unlike the investigations upon which former classification systems were based, the small sample sizes of these studies and their lack of prospective data validation limit the application of the novel taxonomy systems that they are used to develop. Thus, several challenges must be overcome to fully understand and realize the substantial clinical impact of molecular classification and development of precision therapies. Currently, the inherently high heterogeneity of the gastric tumor restricts overall treatment from reaching its ideal effect. To overcome the molecular heterogeneity across the various subtypes of GC when related clinical trials are performed, all GC subtypes should be considered and specific protocols developed for each trial. In the Personalized Antibodies from Gastro-Esophageal Adenocarcinoma (PANGEA) study, an innovative umbrella clinical trial, patients are assigned to different treatment arms by matching a specific drug to a specific GC molecular characteristic [115].

Considering that tumor mutation profiles can evolve over time and respond to comprehensive treatment, the adaptive design of this trial could allow for modifications to certain aspects during the investigation.

## Conclusion

Matching the right drug with the right patient at the right time could greatly improve the curative effect. Precise treatment and refined classification of GC remain ongoing endeavors. Relevant tests on patient-derived tumor xenografts (PDX) and genetically engineered mouse (GEM) models will aid in validating genomic alterations in the molecular subtypes of GC and facilitating drug and biomarker development. The development of novel therapies combining immunotherapy, cytotoxic chemotherapeutic agents, and molecularly targeted therapy is expected to offer durable clinical benefit and maximize the survival of patients with GC.

## Data Availability

No data was used for the research described in the article.
